# Draft Genome Sequence and Annotation of Pseudomonas carnis Strain 20TX0167, Isolated from an Onion (Allium cepa)

**DOI:** 10.1128/mra.01051-22

**Published:** 2023-01-16

**Authors:** Manzeal Khanal, Subas Malla

**Affiliations:** a Department of Horticultural Sciences, Texas A&M University, College Station, Texas, USA; b Texas A&M AgriLife Research and Extension Center, Uvalde, Texas, USA; Loyola University Chicago

## Abstract

The draft genome sequence of Pseudomonas carnis strain 20TX0167, isolated from a cold stored onion bulb, is described here. A comparative genomic study against the type strain of this species, B4-1^T^, revealed differences in some genetic aspects.

## ANNOUNCEMENT

Pseudomonas carnis is a bacterium originally isolated from a spoiled meat sample kept in cold storage (1). *P. carnis* strain 20TX0167 was isolated from a rotting onion bulb stored at 4°C for about a month, following a protocol described earlier (2). Briefly, a 5-mm section, comprising diseased and healthy tissue, was macerated in 100 μL sterile water. A pure culture was obtained after successive culturing from a single colony in nutrient agar (NA) medium. The strain grew well on NA medium at 4°C to 30°C but not at 35°C. Its pathogenicity was tested by inoculating 10-μL and 0.5-mL bacterial suspensions (10^8^ CFU/mL) onto a detached scale of red onion and into a yellow onion bulb, respectively, and incubated at 25°C, as described earlier (3). The strain produced a very mild necrosis reaction on the onion scale and bulb assay.

**FIG 1:**
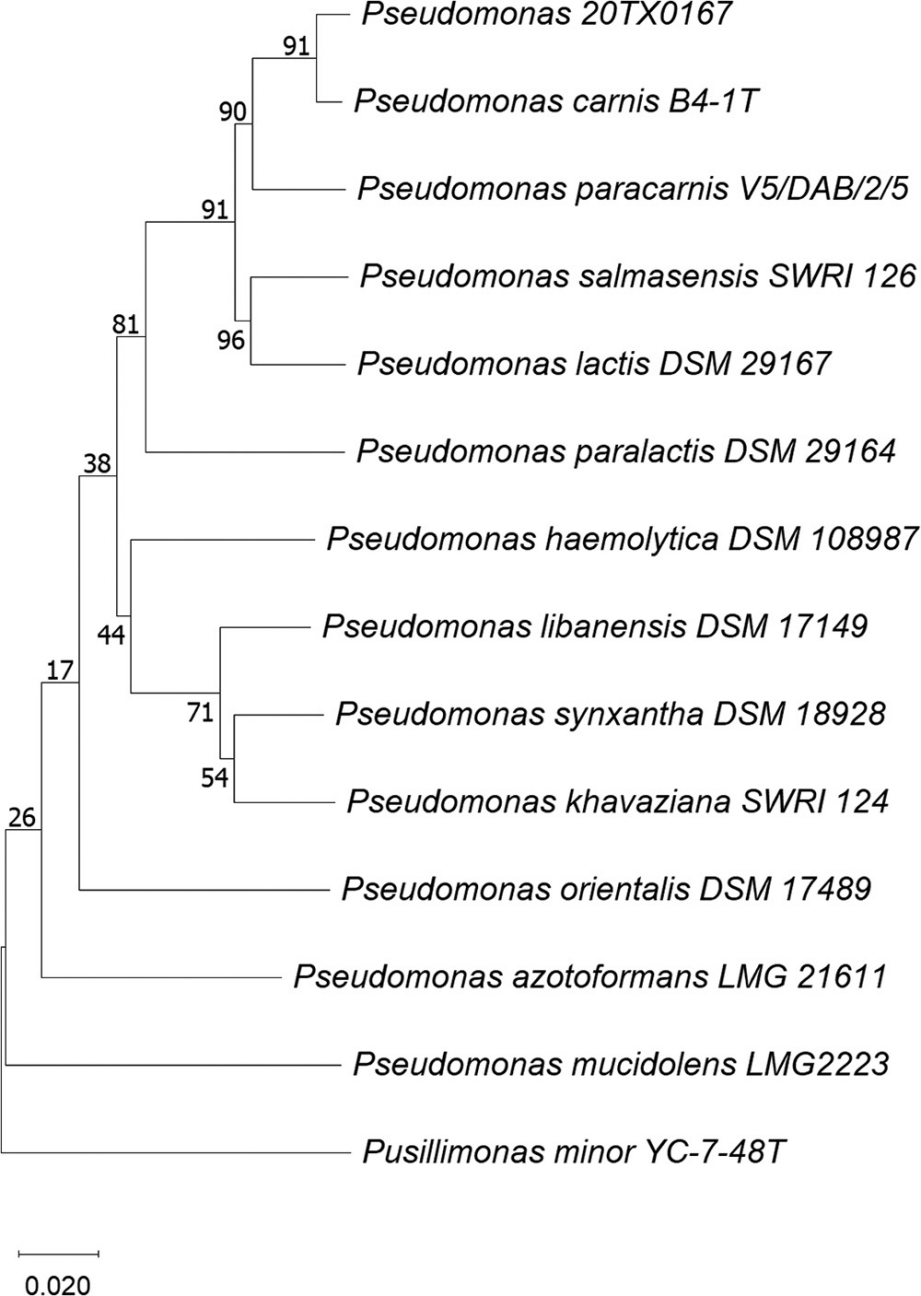
Phylogenetic tree showing the relationships between strain 20TX0167 and closely related species. The phylogenetic tree was drawn using TYGS, and the values at the branching nodes represent bootstrap values based on 100 replicates.

Strain 20TX0167 genomic DNA was extracted from an overnight culture grown on NA medium at 22°C using a DNeasy Power Soil kit (Qiagen), and the genome was sequenced by SeqCenter (Pittsburgh, PA). Sample libraries were prepared using the Illumina DNA prep kit and IDT 10-bp unique dual indexes (UDI) and sequenced on an Illumina NextSeq 2000 platform, producing 2 × 150-bp paired-end reads at a depth of 150×. The data consisted of a total of 3,771,302 reads covering 520,594,806 bp, with a quality score of >Q30. Demultiplexing, quality control, and adapter trimming were performed using BCL Convert v3.9.3 (https://emea.support.illumina.com/sequencing/sequencing_software/bcl-convert.html). The unprocessed reads were paired, trimmed, normalized, and assembled into a draft genome sequence using the Geneious Prime v2021.1.1 functions to select paired reads, run BBDuk, error correct and normalize the reads, and *de novo* assemble the sequences, respectively (4). Default settings were used for all software unless otherwise specified. TYGS (5) was used to compute the digital DNA-DNA hybridization (dDDH) values and draw a phylogenetic tree to compare the strain to closely related species (Fig. 1). The average nucleotide identity (ANI) was calculated using EzBioCloud (6). The dDDH (formula d4), ANI, and G+C difference between our strain and *P. carnis* B4-1^T^ (GenBank genome accession number GCF_902329575.1) were 87.1%, 98.4%, and 0.28%, respectively. The genome sequences, submitted to GenBank (7), were annotated using the NCBI Prokaryotic Genome Annotation Pipeline (PGAP) v6.2 (8), and the summary statistics are shown in Table 1. Using the Rapid Annotation using Subsystems Technology (RAST) online service and the SEED viewer, annotation and comparison of the gene-associated features were carried out for strain 20TX0167 and *P. carnis* B4-1^T^ (9, 10). Out of 1,934 genes with defined functions, 1,892 were common to both, indicating their close resemblance. Among the remaining 42 genes, 20 were found only in 20TX0167, and 22 were found only in B4-1^T^. These genes among the two strains represented 31 unique subsystems; 16 were present only in 20TX0167, and 15 were present only in B4-1^T^ (Table 1). A deeper investigation into their roles could provide an insightful understanding of the adaptation of such strains in cooler spoilage conditions, which may help us tackle the problem of produce loss.

**TABLE 1:** Summary of the genome annotation of *P. carnis* 20TX0167, carried out using the NCBI Prokaryotic Genome Annotation Pipeline and the RAST SEED viewer

Type of data	Characteristic	Value^*a*^
Sequencing metrics	GenBank accession no.	JANQAQ000000000
	Genome size (bp)	6,009,690
	DNA G+C content (%)	60.09
	No. of contigs	46
	Contig *N*_50_ (bp)	245,600
	Contig *L*_50_	7
	Total no. of genes	5,464
	Total no. of CDSs^*b*^	5,383
	No. of CDSs (with protein)	5,297
	No. of genes (RNA)	81
	No. of rRNAs (5S, 16S, 23S)	7, 3, 6
	Complete	7, 1, 1
	Partial	0, 2, 5
	No. of tRNAs	61
	No. of ncRNAs^*c*^	4
	Total no. of pseudogenes	86
Annotated subsystems^*d*^	Chorismate^*e*^	+
	Methionine biosynthesis	+
	Glycine and serine utilization	−
	Pentose phosphate pathway	+
	Glycerate metabolism	−
	Carbohydrates (VC0266)	−
	PFGI-1-like cluster 1	+
	Clustering-based subsystem (316273.3.peg.2378)	−
	NAD and NADP cofactor biosynthesis global	−
	DNA topoisomerases, type I, ATP-independent	+
	Restriction-modification system	+
	Fatty acid biosynthesis FASII	+
	Type 1 pili (mannose-sensitive fimbriae, gamma-fimbriae)	+
	Ton and Tol transport systems	+
	Beta-fimbriae	−
	Flagellum in Campylobacter	+
	Flagellum	−
	Amidase clustered with urea and nitrile hydratase functions	+
	Phage capsid proteins	−
	Phage lysis modules	−
	Phage tail proteins	−
	Potassium homeostasis	+
	Ribosome LSU^*f*^ bacterial	−
	N-linked glycosylation in bacteria	−
	Toxin-antitoxin replicon stabilization systems	+
	MazEF toxin-antitoxin (programmed cell death) system	−
	Terminal cytochrome *d* ubiquinol oxidases	+
	Group II intron-associated genes	+
	Bacterial transcription factors	−
	Glutaredoxins	−
	Multidrug resistance efflux pumps	+

a +, present in strain 20TX0166 but absent from *P. carnis* B4-1^T^; −, absent from strain 20TX0166 but present in *P. carnis* B4-1^T^.

b CDSs, coding DNA sequences.

c ncRNAs, noncoding RNAs.

d Highlighted genomic differences of classified subsystem features.

e Intermediate for synthesis of tryptophan, PAPA antibiotics, PABA, 3-hydroxyanthranilate, and more.

f LSU, large subunit.

### Data availability.

The draft genome shotgun sequences for *P*. *carnis* strain 20TX0167 have been deposited at GenBank, and the accession number is JANQAQ000000000. The SRA accession number is SRR21319267.
